# Psychometric Properties of the Dutch Short Form of the Five‐Factor Narcissism Inventory

**DOI:** 10.1002/pmh.70090

**Published:** 2026-06-26

**Authors:** Etelka J. Harmsen, Peter M. ten Klooster, Joshua D. Miller, Donald R. Lynam, Farid Chakhssi

**Affiliations:** ^1^ Stichting Dimence Groep Deventer the Netherlands; ^2^ Department of Psychology, Health and Technology University of Twente Enschede the Netherlands; ^3^ Department of Psychology University of Georgia Athens Georgia USA; ^4^ Department of Psychological Sciences Purdue University West Lafayette Indiana USA

**Keywords:** cross‐cultural adaptation, factor analysis, Five‐Factor Narcissism Inventory–Short Form (FFNI‐SF), narcissism, psychometric properties, trifurcated model of narcissism, validation

## Abstract

The Five‐Factor Narcissism Inventory–Short Form (FFNI‐SF) is specifically developed to assess narcissism at the two‐ (grandiose and vulnerable narcissism) and three‐factor level (agentic extraversion, self‐centered antagonism, and narcissistic neuroticism). To date, however, no psychometrically validated Dutch language version has been available in research or clinical settings. This study developed and validated the Dutch version of the 60‐item FFNI‐SF (FFNI‐SF‐NL), examining its reliability, factor structure, and convergent and discriminant validity following a preregistered analysis plan. A sample of 223 adult Dutch‐speaking participants, comprising 80 patients with personality disorders or individuals receiving mental health care and 143 non‐clinical participants, completed the FFNI‐SF‐NL. While strict confirmatory factor analysis showed poor model fit for the proposed two‐ and three‐factor models, subsequent exploratory factor analysis and exploratory structural equation modeling supported a three‐factor structure that closely replicated the modern trifurcated conceptualization of narcissism. The FFNI‐SF‐NL vulnerable and grandiose composites and agentic extraversion, self‐centered antagonism, and narcissistic neuroticism factors demonstrated adequate to good reliability and strong external construct validity, with correlations between its dimensions and other narcissism measures, personality traits, and psychological distress aligning closely with theoretical expectations specified a priori. Clinical participants showed higher vulnerable narcissism and narcissistic neuroticism scores, whereas non‐clinical participants showed higher grandiose narcissism and agentic extraversion scores, providing preliminary support for the clinical relevance of distinguishing FFNI‐SF‐NL narcissism dimensions. These results support the FFNI‐SF‐NL as a reliable and valid multidimensional measure capturing nuanced expressions of narcissistic traits in both clinical and non‐clinical Dutch‐speaking populations, facilitating future research and clinical practice.

In contemporary psychological literature, narcissism is often conceptualized as a multidimensional construct that encompasses at least two distinguishable core dimensions: grandiose narcissism and vulnerable narcissism (Cain et al. 2008; Wink 1991). These two dimensions of narcissism exhibit distinct nomological networks with different interpersonal behaviors, emotional patterns, and personality characteristics (Miller et al. 2011). Grandiose narcissism, the prototypical manifestation of narcissism (Lynam and Widiger 2001; Miller et al. 2018), is associated with grandiosity, exploitativeness, a domineering interpersonal style, and high self‐esteem. By contrast, vulnerable narcissism is associated with hypersensitivity, defensiveness, anxiety, mistrust, and emotional vulnerability (Sherman et al. 2015).

Recent factor‐analytic studies have shown that these core dimensions of narcissism can be better understood as being composed of three factors: agentic extraversion, self‐centered antagonism, and narcissistic neuroticism (Crowe et al. 2019; Krizan and Herlache 2018; Miller et al. 2017). This three‐factor conceptualization provides a more nuanced understanding of what is shared across the core dimensions of narcissism and what is unique to grandiose and vulnerable narcissism (Miller et al. 2016, 2017). Specifically, agentic extraversion is strongly related to grandiose narcissism, whereas narcissistic neuroticism is more related to vulnerable narcissism. Self‐centered antagonism, however, is common to both dimensions of narcissism, although it manifests differently across the two dimensions. Three factor models of narcissism appear to be the best way to represent this nuanced, multidimensional construct (Miller et al. 2021).

The Five‐Factor Narcissism Inventory (FFNI; Glover et al. 2012) was specifically developed to assess the specific traits related to grandiose and vulnerable dimensions of narcissism, as well as narcissistic personality disorder. Grounded in the five‐factor model of personality, the FFNI consists of 148 items that assess 15 traits, which were selected based on expert ratings and meta‐analyses. Rationally derived grandiose and vulnerable narcissism composites can be used, which show excellent correspondence with experts' ratings of their respective nomological network (Miller et al. 2014), or empirically derived trifurcated narcissism dimensions (Miller et al. 2016). A shorter version of the inventory, the 60‐item FFNI short form (FFNI‐SF), was later developed and validated using FFNI data collected among four diverse non‐clinical and clinical samples (Sherman et al. 2015). The four‐item subscales retained acceptable reliability and exploratory factor analysis of the 15 subscale scores provided consistent support for a three‐factor solution, consisting of self‐centered antagonism (eight traits), narcissistic neuroticism (three traits, with a secondary loading of reactive anger) and agentic extraversion (four traits), identical to the original long‐form subscales. Correlations with a variety of criterion measures, including basic personality dimensions, other measures of grandiose and vulnerable narcissism, and indicators of externalizing and internalizing psychopathology were similar to those of the long version (Sherman et al. 2015). Grandiose (acclaim seeking, grandiose fantasies, authoritativeness, exhibitionism, lack of empathy, thrill seeking, exploitativeness, arrogance, entitlement, indifference and manipulativeness) and vulnerable (cynicism/distrust, need for admiration, reactive anger, and shame) composite scores also showed very similar correlational profiles across the short‐ and long‐form versions. Several studies have since consistently confirmed the factor structure and psychometric qualities of the FFNI‐SF across diverse languages and cultures (Bahrami and Safarloo 2023; Fossati et al. 2018; Imbeault‐Nepton et al. 2024; Jauk et al. 2023; Papageorgiou et al. 2022). However, none of these studies included a Dutch‐language version or examined its performance in a mixed clinical and community sample.

The FFNI and its short form have proven effective in capturing the core features of both grandiose and vulnerable narcissism and to provide a more nuanced understanding of the narcissistic spectrum, revealing how shared and unique factors contribute to different expressions of narcissism. Examining these dimensions across clinical and non‐clinical populations may further clarify the external validity and clinical applicability of the trifurcated model of narcissism. To date, however, no validated Dutch language version is available for use in clinical and non‐clinical settings. The present study aimed to develop a Dutch version of the Five‐Factor Narcissism Inventory Short Form (FFNI‐SF‐NL) and to examine its psychometric properties in a sample of patients with personality disorders and general population subjects. Given that the trifurcated model of narcissism has emerged as a leading integrative framework for conceptualizing the structure of narcissism, examining the validity of instruments grounded in this model across different cultural and clinical contexts is essential. In addition to evaluating the psychometric properties of the FFNI‐SF‐NL, the present study also explored whether the narcissism dimensions assessed by the instrument could meaningfully differentiate between clinical and non‐clinical participants, thereby providing further evidence for the clinical relevance and external validity of the trifurcated model. Specifically, we investigated the composite reliability, factor structure, and convergent and discriminant validity of the FFNI‐SF‐NL. Based on the reviewed literature, we hypothesized that the 15 FFNI‐SF‐NL subscales would fit both a correlated two‐factor model (vulnerable and grandiose narcissism) and a hierarchical three‐factor model (self‐centered antagonism, narcissistic neuroticism, and agentic extraversion) with one higher‐order overall narcissism dimension. The two‐factor model scores were hypothesized to show strong convergent validity with external criterion measures of grandiose and vulnerable narcissism. For the three‐factor model the agentic extraversion scores were expected to be strongly positively related to general big‐five personality measures of extraversion, self‐centered antagonism strongly negatively related to agreeableness, and narcissistic neuroticism strongly positively related to neuroticism. Additionally, a priori hypotheses were formulated with respect to the convergent and discriminant validity with specific personality traits and aspects of psychological distress.

## 1 Method

The primary goal of the present study was to develop and validate the Dutch short form of the Five‐Factor Narcissism Inventory (FFNI‐SF‐NL). To ensure a rigorous and transparent research process, the primary hypotheses and methodological approach for the present study were preregistered before commencing data collection (Harmsen et al. 2024). The study received ethical approval from the Behavioural, Management and Social Sciences (BMS) Ethics Committee at the University of Twente (approval number: 231462). Informed consent was obtained from all participants prior to data collection through the online survey.

### 1.1 Cross‐Cultural Translation of the FFNI‐SF‐NL

As a first step, the original English version of the FFNI‐SF was translated into Dutch following established forward–backward translation procedures (Guillemin et al. 1993). The items from the English version of the measures were translated into Dutch independently by two of the authors (EJH and FC). Next, the two authors discussed differences in translations until consensus was reached. The consensus version was then back translated into English by a native speaker blinded to the original version. The back translated version was sent to two of the original developers of the FFNI‐SF (JM and DL), who approved the translation after some minor adjustments. The prefinal FFNI‐SF‐NL was pretested in five persons (three patients, three men, age range 26–52 years). Pretests were conducted using the three‐step test interview method (Hak et al. 2008). Based on the results, no additional changes were made.

### 1.2 Participants

Two convenience samples were recruited to complete the FFNI‐SF‐NL along with other selected standardized self‐report measures in a cross‐sectional online survey study. Participants (*n* = 315) were Dutch‐speaking adults recruited via three sources: individuals receiving care at the Center for Personality Disorders of Dimence in Almelo, the Netherlands (*n* = 97), undergraduate students from the University of Twente (*n* = 95), and community participants recruited through word of mouth (*n* = 123).

### 1.3 Procedure

All participants completed an online survey created using Qualtrics survey software. The survey included a question to determine whether they were currently receiving mental health care. Based on responses to this item, participants were categorized into either the clinical or non‐clinical group. Individuals who answered “Yes” or “Less than a year ago” were assigned to the clinical sample. Those who answered “No” or “More than a year ago” were assigned to the non‐clinical sample.

In total, 315 individuals started the survey in Qualtrics. Of these, eight participants (all from the clinical group) did not provide consent to use their data, and seven clinical participants discontinued the survey immediately after giving informed consent; these 15 individuals were excluded from all analyses. An additional 74 participants were excluded because they discontinued the survey early and provided only partial sociodemographic information. These participants included 32 patients, 16 students, and 26 community participants. Finally, three participants were excluded due to indications of careless responding, including one patient, one student, and one community participant. Careless responding was defined as either responding in less than 1 s per question or providing the same response to all items (e.g., selecting “neither agree nor disagree” for every question).

The final total sample consisted of 223 participants. Based on self‐reported engagement with mental health care services, 80 participants (35.9%) were classified as clinical and 143 (64.1%) as non‐clinical participants. Across the total sample, 78 participants (35.0%) were recruited from a university participant pool, 49 (22.0%) from the personality disorders treatment center, and 96 (43.0%) through word‐of‐mouth in the general community.

Within the clinical group, 46 individuals (57.5%) were patients currently receiving treatment, 21 (26.3%) were university students, and 13 (16.3%) were community participants who reported current or recent mental health care usage. Within the non‐clinical group, 57 participants (39.9%) were students, 83 (58.0%) were community participants, and three (2.1%) were patients who reported no recent engagement with mental health care.

The mean age of the total sample was 32.7 years (SD = 13.4, range = 18–72 years). The majority of participants identified as female (71.7%), had attained a high level of education (73.1%), and were engaged in paid employment (48.4%). Additional demographic characteristics, as well as the descriptive statistics for the measures are reported in Table 1 stratified by participant group (clinical vs. non‐clinical). As would be expected, the clinical group reported higher levels of psychological distress (DASS‐total: *M* = 24.73, SD = 14.54) than the non‐clinical group (*M* = 11.02, SD = 11.74). With respect to the FFNI‐SF‐NL scores, the confidence intervals around Cohen's *d* between‐group effect sizes (Cohen 1988) indicated statistically significant differences on grandiose narcissism, vulnerable narcissism, agentic extraversion, and narcissistic neuroticism. Specifically, the clinical group reported higher levels of vulnerable narcissism and narcissistic neuroticism, whereas the non‐clinical group scored higher on grandiose narcissism and agentic extraversion. Between‐group differences were large (*d* ≥ 0.8) for vulnerable narcissism, medium (*d* ≥ 0.5) for narcissistic neuroticism, and small (*d* < 0.3) for grandiose and agentic extraversion.

**TABLE 1:** Descriptive statistics of the clinical and non‐clinical samples.

Variable	Clinical sample (*n* = 80)	Non‐clinical sample (*n* = 143)	*d* (95% CI)
Age, *M* (SD)	33.56 (12.48)	32.29 (13.96)	0.10 (−0.18 to 0.37)
Gender, *n* (%)			
Male	26 (32.50%)	32 (22.38%)	
Female	51 (63.75%)	109 (76.22%)	
Other	3 (3.75%)	2 (1.40%)	
Marital status, *n* (%)			
Married/registered partnership	22 (27.50%)	48 (33.57%)	
Divorced	6 (7.50%)	8 (5.59%)	
Widowed	2 (2.50%)	0 (0.00%)	
Never married	50 (62.50%)	87 (60.84%)	
Educational level, *n* (%)^a^			
Low	5 (6.25%)	1 (0.70%)	
Intermediate	37 (46.25%)	17 (11.89%)	
High	38 (47.50%)	125 (87.41%)	
Work status, *n* (%)			
Paid employment	30 (37.50%)	78 (54.55%)	
Student	24 (30.00%)	57 (39.86%)	
Other (unemployed, retired)	26 (32.50%)	8 (5.59%)	
FFNI‐SF, *M* (SD)			
Overall narcissism	149.35 (21.17)	148.19 (22.95)	0.05 (−0.22 to 0.33)
Grandiose	97.19 (18.92)	104.26 (19.48)	−0.37 (−0.64 to −0.09)
Vulnerable	52.16 (10.33)	43.91 (8.50)	0.90 (0.61–1.18)
Self‐centered antagonism	69.99 (14.71)	69.12 (14.37)	0.06 (–0.21 to 0.33)
Agentic extraversion	42.40 (9.54)	45.69 (8.90)	−0.36 (−0.64 to −0.08)
Narcissistic neuroticism	44.66 (8.79)	38.92 (6.98)	0.75 (0.47–1.03)
NPI, *M* (SD)			
Leadership/authority	10.14 (4.92)	11.57 (4.67)	−0.30 (−0.58 to −0.03)
Grandiose/exhibitionism	12.30 (13.15)	13.15 (4.97)	−0.16 (−0.44 to 0.11)
Entitlement/exploitativeness	12.58 (4.97)	12.48 (3.75)	0.02 (−0.25 to 0.30)
Total score	35.01 (12.58)	37.20 (11.66)	−0.18 (−0.46 to 0.09)
HSNS, *M* (SD)			
Oversensitivity to judgment	5.07 (1.26)	3.99 (1.14)	0.91 (0.63–1.20)
Egocentrism	3.18 (1.07)	2.73 (0.99)	0.44 (0.16–0.72)
Average score	4.12 (1.00)	3.34 (0.95)	0.81 (0.52–1.09)
NARQ, *M* (SD)			
Admiration	1.66 (0.87)	1.90 (0.92)	−0.27 (−0.54 to 0.01)
Rivalry	1.76 (0.94)	1.86 (0.82)	−0.11 (−0.39 to 0.16)
Average score	1.71 (0.85)	1.88 (0.77)	−0.21 (−0.49 to 0.06)
BFI, *M* (SD)			
Extraversion	3.04 (0.76)	3.36 (0.65)	−0.46 (−0.74 to −0.18)
Neuroticism	3.67 (0.69)	2.84 (0.66)	1.24 (0.94–1.53)
Openness to experience	3.44 (0.60)	3.49 (0.57)	−0.09 (−0.36 to 0.18)
Conscientiousness	3.34 (0.71)	3.51 (0.58)	−0.26 (−0.54 to 0.01)
Agreeableness	3.54 (0.62)	3.63 (0.52)	−0.16 (−0.43 to −0.11)
DASS, *M* (SD)			
Total psychological distress (DASS‐Total)	24.73 (14.54)	11.02 (11.74)	1.07 (0.78–1.36)

*Note: N* = 223 (*n* = 80 for clinical sample and 143 for nonclinical sample). *M* = mean, SD = standard deviation, *n* = sample size, *d* = Cohen's *d* with 95% confidence interval.

Abbreviations: BFI = Big Five Inventory; DASS = Depression Anxiety Stress Scales; FFNI‐SF = Five‐Factor Narcissism Inventory–Short Form; HSNS = Hypersensitive Narcissism Scale; NARQ = Narcissistic Admiration and Rivalry Questionnaire; NPI = Narcissistic Personality Inventory.

^a^ Educational level refers to the highest level of education completed.

### 1.4 Five‐Factor Narcissism Inventory Short Form (FFNI‐SF‐NL)

The FFNI‐SF‐NL is a 60‐item self‐report measure of 15 traits related to grandiose and vulnerable narcissism, as well as narcissistic personality disorder (NPD). Vulnerable narcissism can be scored as the sum of distrust, need for admiration, reactive anger, and shame. Grandiose narcissism can be scored using the sum of the remaining scales. The FFNI–SF also yields scores for three empirically derived factors: self‐centered antagonism (sum of manipulativeness, exploitativeness, entitlement, lack of empathy, arrogance, reactive anger, distrust, and thrill seeking), agentic extraversion (sum of acclaim seeking, authoritativeness, grandiose fantasies, and exhibitionism), and narcissistic neuroticism (sum of shame, indifference [reversed], and need for admiration) as suggested by Miller et al. (Miller et al. 2016). Items are measured on a 5‐point Likert scale ranging from 1 (*disagree strongly*) to 5 (*agree strongly*). Cronbach's α values for the dimensions of the original FFNI‐SF have consistently been reported as good to excellent across previous studies (Fossati et al. 2018; Miller et al. 2016; Rogoza et al. 2021). In the original validation study, the higher order dimensions demonstrated strong internal consistency: grandiose narcissism (α = 0.94), vulnerable narcissism (α = 0.85), agentic extraversion (α = 0.90), self‐centered antagonism (α = 0.92), and narcissistic neuroticism (α = 0.88) (Miller et al. 2016).

### 1.5 Construct Validity Measures

#### 1.5.1 Narcissistic Personality Inventory‐13 (NPI‐13)

The NPI‐13 is a 13‐item self‐report measure related to grandiose narcissism (Barelds and Dijkstra 2010; Raskin and Hall 1979). The NPI‐13 has three subscales (leadership–authority [LA], grandiose‐exhibitionism [GE], and entitlement‐exploitativeness [EE]) and can also provide a total score indexing the subject's overall level of grandiose narcissism (Gentile et al. 2013). Items are measured on a 7‐point Likert scale ranging from 1 (*disagree strongly*) to 7 (*agree strongly*). In this study, composite reliability as measured by McDonald's omega was good for the total score of grandiose narcissism (*ω* = 0.86), fair for the LA (*ω* = 0.79) and GE (*ω* = 0.75) subscales, and poor for the EE subscale (*ω* = 0.59).

#### 1.5.2 Hypersensitive Narcissism Scale (HSNS)

The HSNS is a 10‐item self‐report measure of vulnerable narcissism (de Bruin et al. 2017; Hendin and Cheek 1997). The HSNS‐10 has two subscales: oversensitivity to judgment (OJ) and egocentrism (EC), as well as a total score indexing the subject's overall level of vulnerable narcissism (Fossati et al. 2009). Items are measured on a 7‐point Likert scale ranging from 1 (*disagree strongly*) to 7 (*agree strongly*). In this study, reliability was good for the total vulnerable narcissism scale (*ω* = 0.85) and the OJ subscale (*ω* = 0.86), while it was fair for the EC subscale (*ω* = 0.74).

#### 1.5.3 Narcissistic Admiration and Rivalry Questionnaire (NARQ)

The Narcissistic Admiration and Rivalry Questionnaire (NARQ) is a six‐item self‐report measure designed to assess both agentic (admiration) and antagonistic (rivalry) aspects of grandiose narcissism (Back et al. 2013). Additionally, a total score can be computed for the overall level of grandiose narcissism. Items are measured on a 6‐point Likert scale ranging from 1 (*disagree strongly*) to 6 (*agree strongly*). In this study, reliability was good for the total grandiose narcissism scale (*ω* = 0.85) and the admiration subscale (*ω* = 0.81), while it was fair for the rivalry subscale (*ω* = 0.75).

#### 1.5.4 Five‐Factor Model Personality Traits (BFI)

To assess the basic personality traits of extraversion, neuroticism, openness to experience, agreeableness, and conscientiousness, we used the 44‐item Big Five Inventory (Denissen et al. 2008; John and Srivastava 1999). Participants rated their agreement with each item on a 5‐point response scale ranging from 1 (*disagree strongly*) to 5 (*agree strongly*). In this study, the reliability for the subscales Extraversion (ω = 0.85), Neuroticism (ω = 0.87), and Conscientiousness (*ω* = 0.81) was good and for the subscale Openness to Experience (*ω* = 0.76) and Agreeableness (*ω* = 0.74) fair.

#### 1.5.5 Depression, Anxiety, and Stress (DASS)

The Depression, Anxiety, and Stress Scale (DASS) is a 21‐item self‐administered questionnaire designed to measure the magnitude of three negative emotional states: depression, anxiety, and stress (P. F. Lovibond and Lovibond 1995; S. H. Lovibond and Lovibond 2011). The DASS‐Depression focuses on reports of low mood, motivation, and self‐esteem, DASS‐Anxiety on physiological arousal, perceived panic, and fear, and DASS‐Stress on tension and irritability. Participants rated their agreement on a 4‐point response scale ranging from 0 (*did not apply to me at all*) to 3 (*applied to me very much or most of the time*). In the current study, only the total score was used as an index of general psychological distress. The reliability of the total score was excellent (*ω* = 0.96).

### 1.6 Statistical Analyses

To evaluate the psychometric properties of the FFNI‐SF‐NL, a series of statistical analyses were conducted in JASP (version 0.19.3.0). First, the composite reliability of the 15 FFNI‐SF‐NL subscales was examined using McDonald's omega coefficients. Coefficients of < 0.70 were considered unacceptable, coefficients of 0.70–0.79 were considered fair, coefficients of 0.80–0.89 were considered good, and a coefficient of 0.90 or higher was considered excellent (Cicchetti 1994).

Second, structural validity was assessed using maximum likelihood confirmatory factor analysis. Three alternative conceptual models, derived from the original study (Sherman et al. 2015), were tested. Model 1 tested a strict unidimensional structure in which all 15 scales, each consisting of the summed scores of four items, loaded onto a single Narcissism factor (Miller et al. 2016). Model 2 tested the rationally derived structure of two correlated factors: vulnerable and grandiose narcissism. The 15 scales were loaded onto one of the two factors: Vulnerable narcissism (Distrust, Need for admiration, Reactive anger, and Shame) or Grandiose narcissism (Acclaim seeking, Arrogance, Authoritativeness, Entitlement, Exhibitionism, Exploitativeness, Grandiose fantasies, Indifference, Lack of empathy, Thrill seeking, and Manipulativeness) (Miller et al. 2016). Finally, Model 3 tested a hierarchical structure of three first‐order factors (Self‐centered antagonism, Narcissistic neuroticism, and Agentic extraversion), which in turn loaded onto a single second‐order factor. Exploitativeness, Lack of empathy, Thrill seeking, Entitlement, Distrust, Manipulativeness, Arrogance, and Reactive anger loaded onto Self‐centered antagonism. Shame, Low indifference, and need for admiration loaded onto Narcissistic neuroticism (potentially with secondary loadings of Reactive anger). Acclaim seeking, Authoritativeness, Grandiose fantasies, and Exhibitionism loaded onto Agentic extraversion (Fossati et al. 2018; Miller et al. 2016; Sherman et al. 2015). In all models, the scales were constrained to load on one factor only, error terms were not allowed to correlate, and the variance of the factors was fixed to 1. Model fit was determined using several fit indices. Non‐normed fit index (NNFI) and comparative fit index (CFI) values ≥ 0.95 and ≥ 0.90, standardized root mean square residual (SRMR) values ≤ 0.10 and ≤ 0.08, and root mean square error of approximation (RMSEA) values ≤ 0.08 and ≤ 0.06, respectively, were considered indicative of acceptable and good fit (Kline 2011; Vanheule et al. 2012).

Although not specified in the preregistered analytical plan, subsequent exploratory factor analysis (EFA) and exploratory structural equation modeling (ESEM) were used to explore the underlying structure of the scales if none of the conservative CFA models showed adequate fit (even after the addition of theoretically justifiable error term correlations based on modification indices). EFA and ESEM allow for more flexible model estimation and fitting by permitting cross‐loadings and may be more suitable and realistic for complex multidimensional personality measures like the FFNI (Borkenau and Ostendorf 1990; Church and Burke 1994; McCrae et al. 1996). First, principal axis EFA with oblimin rotation was performed to explore whether a two‐factor or three‐factor model appeared to best represent the multidimensional construct of narcissism. Next, we compared the factor loadings of the FFNI–SF‐NL scales for a fixed three‐factor EFA solution with those reported for the FFNI‐SF (Sherman et al. 2015) by computing congruence coefficients (CCs) for the three factors (Tucker 1951). CCs between 0.85 and 0.94 correspond to a fair similarity, while values > 0.95 suggest that the two factors can be considered equal (Lorenzo‐Seva and Ten Berge 2006). Finally, a correlated three‐factor maximum likelihood based ESEM model (Asparouhov and Muthén 2009; Van Zyl and Ten Klooster 2022) was fitted to the 15 scales to assess the fit of the contemporary trifurcated model of narcissism (Miller et al. 2016, 2021; Sherman et al. 2015), with target loadings specified identical to the ones for Model 3 in the CFA analyses. Oblique target rotation was used to maximize the main loadings and minimize cross‐loadings.

Third, convergent and discriminant validity was assessed by means of simple Pearson correlation analyses between the original two‐ and three‐dimensional scores of narcissism and external construct validity measures (Messick 1995). A priori derived hypotheses about the direction and strength of correlations were based on theoretical assumptions and previous empirical findings (Harmsen et al. 2024). Correlations of < 0.3 were considered small, correlations between 0.3 and 0.5 medium, and correlations > 0.5 strong (Cohen 1992). Correspondence between hypothesized and observed correlations was examined by the total proportion of hypotheses met and by means of Pearson profile correlations between hypothesized and observed correlations for each of the FFNI‐SF‐NL factors.

## 2 Results

### 2.1 FFNI‐SF‐NL Scale Descriptive and Reliability Analysis Results

Descriptive statistics and McDonald's omega (*ω*) composite reliability values for FFNI‐SF‐NL subscales and dimensions are summarized in Table 2. Most FFNI‐SF‐NL first‐order scales showed adequate or good composite reliability. Only Acclaim seeking (*ω* = 0.67) and Arrogance (*ω* = 0.69) scored just below the 0.7 cutoff for acceptable reliability. For the different FFNI–SF‐NL second‐order dimensions, fair or good reliability estimates were observed for FFNI‐SF‐NL grandiose narcissism (*ω* = 0.80) and vulnerable narcissism (*ω* = 0.75) and also for FFNI–SF‐NL self‐centered antagonism (*ω* = 0.74), agentic extraversion (*ω* = 0.70), and narcissistic neuroticism (*ω* = 0.78) scales.

**TABLE 2:** Descriptive statistics and reliability estimates for the FFNI‐SF‐NL scales.

Scale	*M*	SD	*ω* (95% CI)	Skewness	Kurtosis
Acclaim seeking	12.21	2.86	0.67 (0.61–0.74)	−0.09	0.12
Arrogance	6.56	2.29	0.69 (0.62–0.76)	1.13	1.22
Authoritativeness	11.64	3.25	0.80 (0.76–0.84)	−0.19	−0.41
Distrust	10.74	3.47	0.80 (0.76–0.84)	0.44	−0.53
Entitlement	7.18	2.80	0.79 (0.75–0.84)	1.09	1.13
Exhibitionism	12.21	3.11	0.74 (0.68–0.79)	−0.14	−0.20
Exploitativeness	7.13	2.74	0.80 (0.75–0.84)	0.92	0.61
Grandiose fantasies	8.45	3.54	0.84 (0.80–0.87)	0.65	−0.32
Indifference	8.83	3.24	0.84 (0.81–0.88)	0.54	−0.22
Lack of empathy	7.70	2.89	0.74 (0.69–0.80)	0.89	0.70
Manipulativeness	10.76	3.20	0.81 (0.77–0.85)	−0.02	−0.55
Need for admiration	12.70	3.36	0.74 (0.69–0.80)	−0.03	−0.33
Reactive anger	10.31	3.12	0.70 (0.63–0.76)	0.20	0.09
Shame	13.12	3.36	0.80 (0.75–0.84)	−0.03	−0.72
Thrill seeking	9.04	3.43	0.82 (0.78–0.85)	0.64	−0.11
Grandiose narcissism	101.72	19.54	0.80 (0.76–0.84)	0.38	0.23
Vulnerable narcissism	46.87	10.00	0.75 (0.69–0.80)	0.43	−0.13
Self‐centered antagonism	69.43	14.46	0.74 (0.69–0.79)	0.52	−0.14
Agentic extraversion	44.51	9.25	0.70 (0.63–0.76)	0.05	0.09
Narcissistic neuroticism	40.98	8.14	0.78 (0.73–0.83)	0.04	−0.47

*Note: N* = 223. *M* = mean; SD = standard deviation; *ω* = McDonald's omega with 95% confidence interval.

### 2.2 Factor Structure

#### 2.2.1 Confirmatory Factor Analysis

The internal construct validity of the FFNI‐SF was evaluated using several confirmatory factor analyses across three models. Model 1 specified a structure with 15 subscales and one higher order factor representing a unified narcissism construct. Model 2 retained the same 15 subscales but included two higher‐order factors corresponding to the grandiosity and vulnerability dimensions of narcissism. Model 3 also preserved the 15 subscales, with agentic extraversion, narcissistic neuroticism, and self‐centered antagonism modeled as higher‐order factors. As shown in Table 3, Models 2 and 3 demonstrated the best but still poor fit across all indices. Examination of the modification indices for the three‐factor model indicated that model fit could be notably improved by allowing several subscales to cross‐load on another factor, such as Distrust on the factor narcissistic neuroticism (MI = 48.27). Furthermore, several significant correlated error terms were suggested between subscales, for example between Manipulativeness and Authoritativeness (MI = 45.01). Despite these indications, such modifications were not implemented due to a lack of theoretical justification for several of the suggested cross‐loadings and error correlations and to preserve model parsimony.

**TABLE 3:** Goodness‐of‐fit statistics for the three models of confirmatory factor analysis.

	χ^2^ (df)	χ^2^ (df)	CFI	NNFI	SRMR	RMSEA (90% CI)
Model 1	702.90 (90)	7.81	0.475	0.388	0.149	0.175 (0.163–0.187)
Model 2	529.55 (89)	5.95	0.623	0.555	0.134	0.149 (0.137–0.161)
Model 3	481.05 (87)	5.53	0.663	0.593	0.131	0.143 (0.130–0.155)

*Note: N* = 223. Model 1: Unidimensional model (15 subscales loading on one narcissism factor). Model 2: Correlated two‐factor model (15 subscales loading on either grandiosity or vulnerability). Model 3: Hierarchical three‐factor model (15 subscales loading on agentic extraversion, narcissistic neuroticism, or self‐centered antagonism, in turn loading on one higher‐order narcissism factor).

#### 2.2.2 Exploratory Factor Analysis

Given the poor overall model fit confirmed through CFA, we conducted an additional EFA using principal axis factoring and oblimin rotation on the 15 FFNI–SF scales, which allow free estimation of factor (cross‐) loadings to explore the observed dimensional structure of the FFNI‐SF.

**FIGURE 1:**
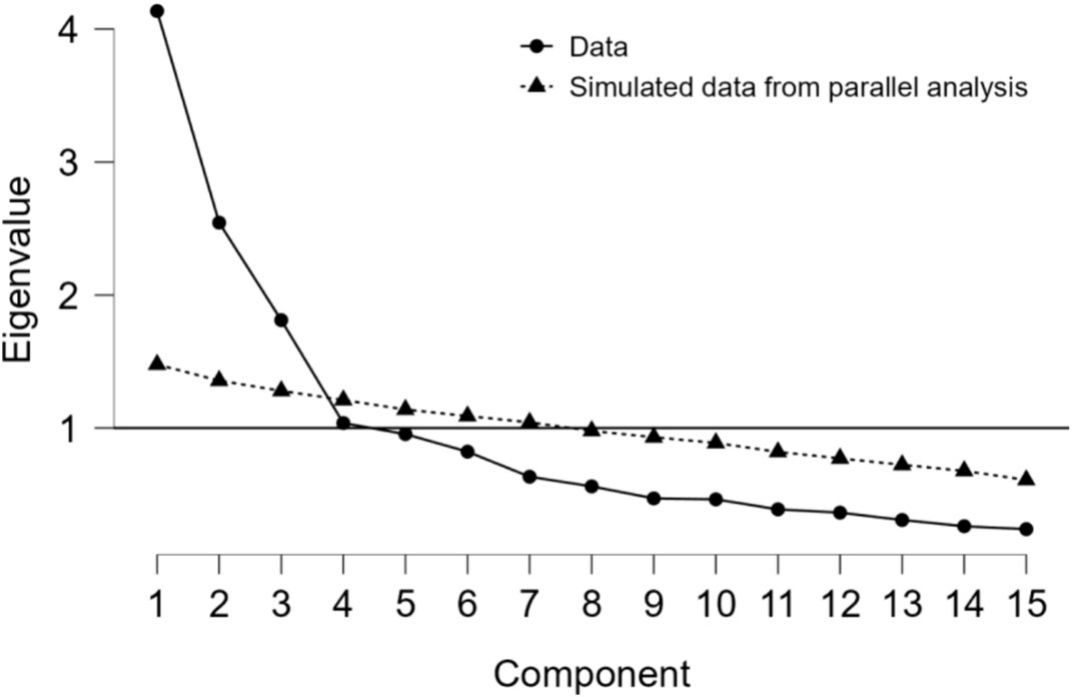
Scree plot of eigenvalues of rotated factors.

Horn's parallel analysis (20 resamplings) was used as the primary criterion to determine the number of factors underlying the 15 subscales. Parallel analysis indicated that the three factors should be retained, as the eigenvalues of the first three extracted factors exceeded those obtained from simulated random data. This suggests that these factors reflect meaningful dimensions, beyond those that would be expected by chance. For completeness, the scree plot and eigenvalues are presented in Figure 1, although these were not used as decision criteria. The scree plot showed a clear break after the third factor, which is consistent with the results of the parallel analysis. Together, the three factors explained 56.7% of the common variance in the subscale scores.

The results of the EFA three‐factor solution are shown in Table 4. Lack of Empathy, Arrogance, Exploitativeness, and Entitlement loaded strongly on Factor 1, which appeared to represent a core antagonistic dimension. Other scales, such as Indifference, Distrust, Grandiose Fantasies, and Reactive Anger, also showed weak to moderate loadings on this factor, but loaded more strongly on other factors. Factor 2 was primarily defined by strong loadings from Grandiose Fantasies, Exhibitionism, Acclaim Seeking, Authoritativeness, Manipulativeness, and Thrill Seeking. Lack of Empathy also showed a weak negative loading on this factor, indicating an inverse, though minor, association. Factor 3 was defined by relatively high loadings from Distrust, Reactive Anger, Need for Admiration, and Shame, as well as a negative loading from Indifference. This dimensional structure largely reflects the previously established three‐factor model, but with some notable differences. Factor 1 closely resembles the trait of Self‐centered antagonism, but with substantial cross‐loadings of Indifference (which loads positively on Self‐centered antagonism and negatively on Narcissistic neuroticism, consistent with the content of these two factors) and Grandiose fantasies (Agentic extraversion), and no substantive loading for Thrill seeking and Manipulativeness. Factor 2 corresponded to Agentic extraversion, with substantive additional loadings for Manipulativeness, as well as a weak loading for Thrill Seeking and a weak negative loading for Lack of Empathy. Factor 3 aligns with Narcissistic neuroticism, but with substantial cross‐loadings of Distrust. Congruence coefficients with the three‐factor solution reported by Sherman et al. (2015) suggested equality for Self‐centered antagonism (CC = 0.97) and Narcissistic neuroticism (CC = 0.95) and fair similarity for Agentic extraversion (CC = 0.88). These results confirm the similarity between the Dutch and original factor structures.

**TABLE 4:** Rotated factor loadings of the 15 FFNI‐SF‐NL scales based on exploratory factor analysis.

FFNI‐SF scales	Factor loading		
	1	2	3
Lack of empathy	0.77	−0.27	
Arrogance	0.69		
Exploitativeness	0.68		
Entitlement	0.59		
Indifference	0.42		−0.48
Distrust	0.40		0.50
Grandiose fantasies	0.35	0.46	
Reactive anger	0.33		0.39
Exhibitionism		0.71	
Acclaim seeking		0.61	
Authoritativeness		0.58	
Manipulativeness		0.58	
Thrill seeking		0.26	
Need for admiration			0.91
Shame			0.74

*Note: N* = 223. The extraction method was principal axis factoring with an oblimin rotation. The threshold for displaying factor loadings was set at 0.25.

To evaluate the convergent validity of the empirically extracted factors, factor scores were saved and correlated with the three a priori FFNI factors (Self‐centered antagonistic, Agentic extraversion, and Narcissistic neuroticism). The results showed that Factor 1 was strongly associated with FFNI‐SF Self‐centered antagonism (*r* = 0.92, *p* < 0.001), Factor 2 with FFNI‐SF Agentic extraversion (*r* = 0.94, *p* < 0.001), and Factor 3 with FFNI‐SF Narcissistic neuroticism (*r* = 0.92, *p* < 0.001), supporting the interpretation of the extracted factor structure.

#### 2.2.3 Exploratory Structural Equation Modeling

The congruence between the EFA results and the contemporary trifurcated model of narcissism prompted further model testing. Specifically, a correlated three‐factor ESEM model was estimated using the 15 scales. Target loadings were specified to be identical to those defined for Model 3 in the CFA analyses in order to directly evaluate the fit of the proposed trifurcated structure. Fit indices of the ESEM model were substantially better than those for the three‐factor CFA model, *χ* ^2^(63) = 212.12, CFI = 0.87, NNFI = 0.79, SRMR = 0.049, RMSEA = 0.103 [90% CI: 0.088–0.118], but still inadequate except for SRMR. Iterative inspection of the modification indices indicated three clear error covariances with MIs around 20 between Authoritativeness and Manipulativeness (MI = 26.92), Authoritativeness and Acclaim Seeking (MI = 21.93), and Distrust and Reactive Anger (MI = 18.72). Allowing their error terms to correlate resulted in overall adequate fit for the three‐factor model, *χ* ^2^(63) = 142.62, CFI = 0.93, NNFI = 0.88, SRMR = 0.042, RMSEA = 0.079 [90% CI: 0.062–0.095], with remaining MIs well below 20. Target factor loadings and observed cross‐loadings were similar to those observed in the three‐factor EFA (Table 5).

**TABLE 5:** Standardized factor loadings of the 15 FFNI‐SF‐NL scales based on exploratory structural equation modeling.

FFNI‐SF scales	Factor loading		
	Self‐centered antagonism	Agentic extraversion	Narcissistic neuroticism
Manipulativeness	**0.26**	0.46	−0.09
Exploitativeness	**0.73**	0.15	−0.05
Entitlement	**0.64**	0.18	−0.05
Lack of empathy	**0.74**	−0.35	−0.14
Arrogance	**0.74**	0.08	−0.10
Reactive anger	**0.34**	0.12	0.28
Distrust	**0.43**	−0.25	0.41
Thrill seeking	**0.23**	0.26	−0.18
Acclaim seeking	0.09	**0.49**	0.03
Authoritativeness	0.05	**0.37**	−0.26
Grandiose fantasies	0.43	**0.40**	0.12
Exhibitionism	−0.03	**0.84**	0.11
Shame	0.10	0.04	**0.68**
Indifference	0.35	−0.13	**−0.54**
Need for admiration	0.07	−0.02	**0.97**

*Note: N* = 223. Results based on maximum likelihood estimation with oblique target rotation. Target loadings are in bold and underlined.

### 2.3 External Construct Validity

Table 6 presents the convergent and discriminant correlations between the various FFNI‐SF factors based on the original (hypothesized) two‐ and three‐factor models and the external criteria. Overall, the FFNI‐SF dimensions exhibited satisfactory convergent and discriminant validity, as evidenced by moderate to strong correlations with criterion measures and conceptually related external constructs and weak correlations with divergent constructs.

**TABLE 6:** Hypothesized and observed correlations between FFNI‐SF‐NL factors and related constructs.

Factor/variable	FFNI‐SF grandiose		FFNI‐SF vulnerable		FFNI‐SF agentic extraversion		FFNI‐SF self‐centered antagonism		FFNI‐SF narcissistic neuroticism	
	Expected	Observed	Expected	Observed	Expected	Observed	Expected	Observed	Expected	Observed
NPI total	> 0.5^c^, ^d^	**0.73**	−0.3–0^c^, ^d^	0.10	> 0.5^b^, ^c^	**0.72**	0.3–0.5^b^; > 0.5^c^	**0.58**	−0.3–0.3^b^, ^c^	**−0.09**
NPI leadership/authority	> 0.5^c^, ^d^	**0.62**	−0.3–0^c^, ^d^	**−0.02**	> 0.5^c^	**0.69**	0.3–0.5^c^	**0.42**	−0.3–0^c^	**−0.13**
NPI grandiose/exhibitionism	0.3–0.5^c^; > 0.5^d^	**0.61**	−0.3–0.3^c^, ^d^	**0.01**	> 0.5^c^	**0.57**	0.3–0.5^c^	**0.45**	−0.3–0^c^	**−0.14**
NPI entitlement/exploitativeness	0.3–0.5^c^; > 0.5^d^	**0.64**	0.0–0.3^d^; 0.3–0.5^b^	**0.32**	0.3–0.5^b^	0.58	> 0.5^c^	**0.64**	0.0–0.3^c^	**0.06**
HSNS total	0.0–0.3^c^, ^d^	**0.15**	> 0.5^c^, ^d^	**0.78**	0.0–0.3^b^, ^c^	**0.11**	0.3–0.5^b^, ^c^	**0.42**	> 0.5^b^, ^c^	**0.64**
HSNS egocentrism	0.0–0.3^a^	0.35	> 0.5^a^	**0.56**	0.0–0.3^a^	**0.19**	0.3–0.5^a^	0.55	0.3–0.5^a^	**0.34**
HSNS oversensitivity	0.0–0.3^a^	−0.04	> 0.5^a^	**0.79**	0.0–0.3^a^	**0.03**	0.0–0.3^a^	**0.22**	> 0.5^a^	**0.75**
NARQ total	> 0.5^a^	**0.68**	0.0–0.3^a^	**0.26**	> 0.5^a^	**0.53**	> 0.5^a^	**0.68**	−0.3–0.3^a^	**−0.01**
NARQ admiration	> 0.5^c^	**0.68**	0.0–0.3^c^	**0.15**	> 0.5^b^, ^c^	**0.54**	0.3–0.5^b^; > 0.5^a^	**0.62**	−0.3–0^b^, ^c^	**−0.08**
NARQ rivalry	> 0.5^c^	**0.55**	0.3–0.5^c^	**0.33**	0.3–0.5^b^, ^c^	**0.41**	> 0.5^b^, ^c^	**0.61**	0.0–0.3^b^, ^c^	**0.07**
BFI extraversion	0.3–0.5^a^	**0.33**	−0.3–0.3^a^	−0.38	> 0.5^b^	0.40	0.0–0.3^b^	**0.08**	−0.3–0.0^b^	−0.38
BFI neuroticism	−0.3–0^a^	**−0.20**	0.3–0.5^a^	0.71	−0.3–0^b^	**−0.15**	0.0–0.3^b^	**0.08**	> 0.5^b^	**0.67**
BFI openness	0.3–0.5^a^	**0.24**	−0.3–0^a^	**−0.06**	0.3–0.5^b^	**0.33**	−0.3–0.3^b^	**0.07**	−0.3–0^b^	**−0.06**
BFI agreeableness	−0.3–0^a^	−0.41	−0.3–0^a^	−0.48	−0.3–0^b^	**−0.22**	< −0.5^b^	**−0.61**	−0.3–0^b^	**−0.10**
BFI conscientiousness	−0.3–0^a^	**−0.16**	−0.3–0^a^	**−0.16**	−0.3–0.3^b^	**0.02**	−0.3–0^b^	**−0.24**	−0.3–0^b^	**−0.07**
DASS total	0.0–0.3^a^	**0.06**	0.3–0.5^a^	0.66	0.0–0.3^a^	**0.01**	0.0–0.3^a^	0.31	0.3–0.5^a^	0.52

*Note: N* = 223. Bold and underlined observed correlations indicate correspondence with a priori expected correlations.

Abbreviations: BFI = Big Five Inventory; DASS = Depression Anxiety Stress Scales; FFNI‐SF = Five‐Factor Narcissism Inventory–Short Form; HSNS = Hypersensitive Narcissism Scale; NARQ = Narcissistic Admiration and Rivalry Questionnaire; NPI = Narcissistic Personality Inventory.

^a^ Based on own hypotheses.

^b^ Based on observed correlation(s) in Rogoza et al. (2021).

^c^ Based on observed correlation(s) in Jauk et al. (2023).

^d^ Based on observed correlation(s) in Glover et al. (2012).

However, several deviations from expectations emerged. Vulnerable narcissism and narcissistic neuroticism showed stronger‐than‐anticipated associations with negative emotionality, as indicated by the DASS scales. In contrast, agentic extraversion was less strongly related to BFI Extraversion than hypothesized. Vulnerable narcissism was more strongly associated with BFI Neuroticism than expected, and both grandiose and vulnerable narcissism showed stronger negative correlations with BFI Agreeableness than anticipated.

In total, 82.5% of the observed correlations were consistent not only in direction but also in magnitude with the a priori hypotheses, indicating strong empirical support for the external construct validity of the FFNI‐SF dimensions of narcissism. Moreover, correlations revealed high similarity between the expected and observed correlation vectors for all dimensions: 0.94 (95% CI: 0.84–0.98) for Grandiose narcissism, 0.89 (95% CI: 0.70–0.96) for Vulnerable narcissism, 0.95 (95% CI: 0.85–0.98) for Agentic extraversion, 0.98 (95% CI: 0.95–0.99) for Self‐centered antagonism, and 0.95 (95% CI: 0.86–0.98) for Narcissistic neuroticism. Together, these results provide strong empirical support for the convergent and discriminant validity of the FFNI‐SF dimensions.

## 3 Discussion

This study aimed to develop and validate a Dutch translation of the Five‐Factor Narcissism Inventory Short Form (FFNI‐SF‐NL). The findings provide psychometric support for the FFNI‐SF‐NL as a measure for assessing multifaceted narcissism in Dutch‐speaking samples. The instrument demonstrated adequate to good composite reliability for its subscales which showed strong convergent and discriminant validity with other measures of narcissism, basic personality traits, and psychological distress. Strict confirmatory factor analysis did not support the hypothesized two‐ or three‐factor models, as is often the case for complex, multidimensional personality inventories (Hopwood and Donnellan 2010). However, a subsequent exploratory factor analysis revealed a three‐factor structure that closely aligns with the intended conceptualization of the agentic extraversion, self‐centered antagonism, and narcissistic neuroticism dimensions.

### 3.1 Reliability of the FFNI–SF‐NL

The 15 narcissistic traits measured by the FFNI–SF–NL, along with the examined higher‐order dimensions, demonstrated predominantly acceptable to strong composite reliability, indicating that each trait and the higher order factors are reliably captured by their respective items. Although the reliability for a few lower order scales (acclaim seeking and arrogance) fell just below the conventional reliability threshold, this is consistent with patterns observed in the original validation of the FFNI‐SF (Miller et al. 2016). However, the composite reliability of the higher order factors remained at least acceptable.

### 3.2 Factor Structure of the FFNI–SF‐NL

CFA model fit indices did not support either the rationally derived two‐factor structure of grandiose and vulnerable narcissism or the empirically derived three‐factor structure of agentic extraversion, self‐centered antagonism, and narcissistic neuroticism (Miller et al. 2016, 2018). Although CFA is often considered the gold standard for testing theorized factor structures, it has several limitations that may be particularly relevant to this study. In CFA, cross‐loadings, while theoretically plausible, are constrained to zero, which likely contributed to the poor model fit. In retrospect, our exclusive reliance on conventional CFA fit in the preregistered analysis plan was likely overly stringent and insufficiently flexible for capturing the structural complexity inherent in a nuanced personality measure such as the FFNI‐SF (Hopwood and Donnellan 2010; Marsh et al. 2010; McCrae et al. 1996). In addition, the poor model fit should be interpreted in light of sample‐related limitations. The relatively small and demographically skewed sample, with predominantly highly educated young women, may have reduced the stability of parameter estimates and restricted variance, thereby obscuring the expected factor structure. Accordingly, conclusions regarding the internal structure should be considered preliminary, and further research using larger and more diverse samples is needed.

Given that the conceptualization of narcissism by Miller et al. (2016) explicitly assumes that different narcissism traits or facets may have a differential impact on narcissism factors, we subsequently performed a non‐preregistered EFA on the 15 subscales. The resulting solution showed a clear and well‐interpretable three‐factor solution with several substantial and plausible cross‐loadings for some of the subscales. Furthermore, congruence coefficients indicated that the factor solution was comparable to the one reported by Sherman et al. (2015). These findings support the structural comparability of the Dutch FFNI‐SF to the original version and also strongly mirrored those found in other international validation studies (Fossati et al. 2018; Papageorgiou et al. 2022; Rogoza et al. 2021). Moreover, the high correlations between the extracted factor scores and the a priori assumed FFNI‐SF factor sum scores indicated that the FFNI‐SF‐NL measures the same core components of agentic extraversion, self‐centered antagonism, and narcissistic neuroticism as the original. Additional ESEM analysis confirmed that the currently proposed three‐factor model (Crowe et al. 2019; Miller et al. 2016, 2021; Sherman et al. 2015) adequately fitted the FFNI‐SF data if minimal cross‐loadings were allowed. This finding corresponds with recent validation studies that also used the more flexible ESEM approach for this reason to test the proposed trifurcated model of narcissism as measured with the FFNI or FFNI‐SF (Imbeault‐Nepton et al. 2024; Jauk et al. 2023; Packer West et al. 2025; Papageorgiou et al. 2022).

### 3.3 External Construct Validity of the FFNI‐SF‐NL

The current findings also provide strong evidence for the external construct validity of the FFNI‐SF‐NL. The observed pattern of associations across multiple criteria measures reflects a high degree of theoretical convergence, both at the two‐factor level (grandiose and vulnerable) and at the three‐factor level (agentic extraversion, self‐centered antagonism, and narcissistic neuroticism). These findings indicate that the FFNI‐SF‐NL successfully captures the multifaceted structure of narcissism as conceptualized within the FFNI framework. Moreover, the alignment with established narcissism measures, relevant Five‐Factor Model traits, and indicators of psychological distress highlights the instrument's nomological validity and clinical utility. The strength and consistency of profile correlations, combined with minimal associations with theoretically unrelated constructs, further support its discriminant validity.

Furthermore, comparisons between the clinical and non‐clinical groups revealed a theoretically consistent pattern of differences in FFNI‐SF‐NL factor scores. The clinical sample reported higher levels of vulnerable narcissism and narcissistic neuroticism, whereas the non‐clinical sample reported higher levels of grandiose narcissism and agentic extraversion. These findings provide additional support for the criterion validity of the FFNI‐SF‐NL and complement the convergent and discriminant validity evidence described above, demonstrating that the instrument can meaningfully differentiate between clinical and community populations.

These findings are also broadly consistent with previous research including clinical populations. For example, a study by Jauk et al. (2023) showed that individuals with mental disorder diagnoses reported elevated levels of vulnerable narcissism and narcissistic neuroticism, a pattern that was also observed in the present study. With regard to self‐centered antagonism, findings appear to depend on the type of clinical population. In Jauk et al. (2023), elevated levels were observed particularly in offender samples, whereas differences were less consistent in general clinical samples. In the present study, no significant differences between the clinical and non‐clinical groups were observed for this dimension, suggesting that antagonistic features may be more characteristic of specific clinical subgroups rather than uniformly elevated across all clinical populations. Taken together, these findings underscore the importance of considering clinical heterogeneity when interpreting narcissism profiles.

### 3.4 Limitations

The present study has several limitations. Although the inclusion of a clinical sample represents a notable strength, its relatively small size limited the ability to conduct meaningful comparisons between clinical and non‐clinical participants. Moreover, the total sample was modest in size and predominantly composed of highly educated young adult women, which restricts the generalizability of the findings to the broader Dutch population. The use of convenience sampling further limits the representativeness of the data. Another limitation concerns the classification of participants into the clinical subsample, which was based on self‐identification rather than verified diagnostic information. This approach may have introduced variability in clinical severity and diagnostic status. Such heterogeneity could attenuate correlations between narcissism scores and clinical outcomes, potentially leading to an underestimation of the instrument's validity. Although the analysis protocol included multiple‐group confirmatory factor analyses to examine measurement invariance across gender and sample type, these analyses were not conducted because none of the tested CFA models showed adequate model fit. As a result, conclusions regarding the comparability of the factor structure across subgroups could not be drawn. In addition, data were exclusively collected via self‐report measures, which may artificially inflate correlations with external measures due to common method variance (Lindell and Whitney 2001). Finally, the cross‐sectional design of the study precludes any conclusions about the temporal stability or predictive validity of the FFNI–SF–NL. Longitudinal research will be essential to assess how narcissistic traits, as measured by the FFNI‐SF‐NL, function over time and across different contexts (Edershile and Wright 2021).

### 3.5 General Implications and Future Directions

This study provides further evidence that the FFNI‐SF can accurately assess narcissism across different languages, cultures, and contexts. The internal construct validity of the three‐factor structure underlying narcissism, supported by the moderate to strong congruence with the contemporary trifurcated model of narcissism, underscores the complexity of narcissism and highlights the importance of a multidimensional approach in both research and clinical practice. Importantly, the present study extends prior work by examining the FFNI‐SF in a Dutch‐speaking sample that included a substantial clinical subsample, enhancing the relevance of the findings for both research and clinical applications in the Netherlands. For clinicians, these findings imply that interventions may need to be tailored to specific narcissistic profiles. For example, a patient presenting with high scores on narcissistic neuroticism might benefit from compassion‐focused therapy or interventions targeting emotion regulation and cognitive distortions, whereas a client high on self‐centered antagonism may require approaches focused on mentalization and perspective‐taking. For researchers, this suggests that treating narcissism as a unitary construct may obscure important differential relationships with external criteria. Future research should therefore continue to explore how these profiles relate to clinically relevant outcomes, such as self‐criticism, shame, and self‐compassion. Moreover, subtle differences in facet loadings across translations highlight the need for continued exploration of how narcissism unfolds in different cultures and contexts. To address the limitations of the current sample and enhance generalizability, future research should aim to replicate these findings in larger, more diverse populations. Furthermore, to overcome the limitations of the cross‐sectional design, longitudinal and multimethod approaches are needed for assessing the temporal stability and predictive validity of the FFNI‐SF‐NL.

In conclusion, this study provides preliminary evidence that the FFNI‐SF‐NL is a reliable and valid instrument for assessing the multidimensional construct of narcissism in Dutch‐speaking populations.

## Funding

The authors have nothing to report.

## Conflicts of Interest

The authors declare no conflicts of interest.

## Data Availability

The data that support the findings of this study are available on request from the corresponding author. The data are not publicly available due to privacy or ethical restrictions.
